# Diagnostics and treatment of orbital myositis

**DOI:** 10.22336/rjo.2022.34

**Published:** 2022

**Authors:** Aida Pidro, Admira Dizdarević, Nina Jovanović, Alma Čerim, Emina Sačak, Ajla Pidro Miokovic

**Affiliations:** * Department of Ophthalmology, “Prim. dr. Abdulah Nakas” General Hospital, Sarajevo, Bosnia and Herzegovina; ** Department of Ophthalmology, Canton Hospital Zenica, Bosnia and Herzegovina; *** Department of Internal Medicine, Canton Hospital Zenica, Bosnia and Herzegovina; **** Policlinic Vukas, Zagreb, Croatia

**Keywords:** orbital myositis, corticosteroids, immunosuppressive therapy

## Abstract

**Objective:** Orbital myositis is a rare clinical condition that involves idiopathic inflammation mostly of extraocular muscles. The purpose of this study was to present a diagnostic and treatment plan of orbital myositis.

**Methods:** A 60-year-old female presented with decreased visual acuity on her left eye, ocular hypertension, restricted and painful left abduction, diplopia, swollen eyelids, and orbital discomfort. MRI, as well as ultrasound, showed enlargement in width of medial rectus muscle. After other diagnoses were excluded, the diagnosis of left orbital myositis was established.

**Results:** She was started on systemic corticosteroid treatment, but each time the steroid dose was tapered she experienced a relapse. Immunosuppressive therapy was introduced and the steroid dose was gradually tapered and excluded. One year after immunosuppressive therapy, the clinical findings improved.

**Conclusion:** The diagnosis of orbital myositis requires detailed examination, laboratory testing and MRI scans of the orbits in order to exclude other diseases with similar clinical findings. The first line treatment option is systemic corticosteroid therapy with additional immunosuppressive therapy if needed.

**Abbreviations:** MRI = magnetic resonance imaging, BCVA = best corrected visual acuity, ENT = ear, nose, throat specialist, CBC = complete blood count, WBC = white blood cell, ESR = erythrocyte sedimentation rate, CRP = C reactive protein, HM = hand motion, TED = thyroid eye disease, SLE = systemic lupus erythematosus, ECG = electrocardiogram, CT = computed tomography

## Introduction

Orbital myositis was first described by Gleason in 1903 as an orbital pseudotumor and in 1930 renamed as benign idiopathic autoimmune inflammatory disease primarily involving one or more extraocular muscles [**1**]. It is a rare disease and is currently considered a subtype of orbital inflammatory syndrome. It usually affects middle-aged women [**2**]. Extraocular muscles differ from skeletal muscles by a smaller unit size and higher motor neuron discharge, higher blood flow and volume of mitochondria fractions that allow inflammatory cells to reach and circulate easier, causing inflammation [**3**].

Usual clinical findings include orbital discomfort, moderate to severe orbital pain, painful diplopia increased with eye movement, exophthalmos, swollen eyelids, conjunctival hyperemia or chemosis [**4**]. Orbit ultrasound can be used for evaluation and a follow up of extraocular muscle enlargement. MRI of the orbits is the best method to evaluate soft tissue that can show the enhancement or the thickening of inflamed extraocular muscles, usually affecting myotendinous insertion as well [**5**].

## Methods

A sixty-year-old female was referred to the Department of Ophthalmology in Canton Hospital Zenica due to her complains of decreased visual acuity, and diplopia with restricted and painful left eye movement. She denied any recent trauma, fever, history of autoimmune and thyroid disease. 

**Fig. 1:**
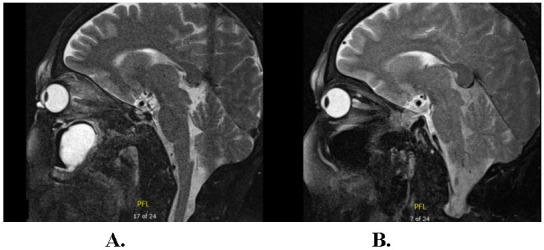
MRI with contrast, sagittal view. **A.** Enlargement of left extraocular muscles with enlargement of their tendon. A maxillary retention cyst was also noted. **B.** Normal size of right eye extraocular muscles

MRI (**Fig. 1**) showed a spindle-shaped mass reaching from posterior globe edge to the orbital apex, partially compromising the optic nerve. Signs of inflammatory process in the left half of the sphenoidal sinus and the left maxillary sinus with larger retention cystic formation were also present. Differential diagnosis included inflammatory pseudotumor. 

Ophthalmological findings on her right eye were unremarkable. However, the examination on her left eye was as follows: BCVA hand motion, normotension, upper and lower eyelid oedema, narrow eyelid rim, pain, restricted abduction, diplopia, conjunctival hyperaemia, reactive pupils, oedematous optic nerve head with unclear borders, restricted abduction in upper and lower temporal gaze in the left eye with a force adduction of the right eye. Left eye ultrasound showed medial rectus muscle enlargement (3.88 mm) with visible Tenon area. 

Additional tests were ordered, together with the reference for rheumatology consult after the completion of immunological examination results, which were all within referenced values, as well as thyroid gland examination. 

Initial diagnosis of left orbital myositis was made and the patient was started on parenteral corticosteroid therapy with methylprednisolone (1 mg/ kg) for 10 days, with a gradual taper. Vitamin D supplement, calcium and proton pump inhibitor were also introduced to decrease the side effect of high dose steroid use. She was given local steroids. Oral antibiotics were also introduced due to pansinusitis, diagnosed by the ENT specialist. Her symptoms resolved gradually over the next few days.

She was released from the hospital as recovered with improved, but still restricted left globe motility, without diplopia, BCVA 0.7 and reduced medial rectus muscle thickness (1.98 mm). She was instructed to continue to take 60 mg oral prednisone daily for 7 days, with a 10 mg tapper per week. As the steroid dose was tapered to 40 mg, our patient came back complaining of orbital pain, swollen eyelids with narrow eyelid rim, severe conjunctival chemosis and hyperemia, restricted left abduction with BCVA 0.5, increased intraocular pressure 41 mmHg and 6 mm left eye proptosis. She was then administered a pulse corticosteroid therapy, 1g for 3 days, followed by a gradual tapper dose of 1 mg/ kg.

**Fig. 2:**
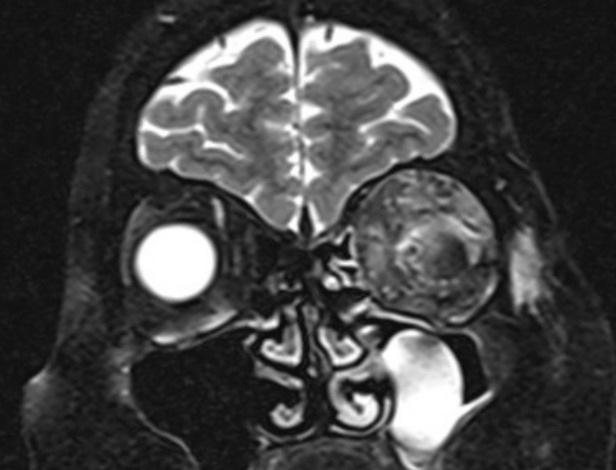
MRI scan, coronal view showing left extraocular muscle enlargement along with the enlargement of lacrimal gland

Urgent neurocranium MRI (**Fig. 2**) showed left globe prominence together with lateral and superior rectus muscle thickening and slight thickening of medial rectus muscle. An increase in the volume of lacrimal gland and suppression of optic nerve inferiorly and medially, with signs of compression, were also observed. 

Since she had experienced exacerbation two times and showed corticosteroid induced chronic obstructive pulmonary disease, increased glucose blood levels followed by the weight gain, it was concluded that she should be started on steroid-sparing agent. After a rheumatology consult and unremarkable lab results (CBC, WBC, urine, kidney, and liver function), immunosuppressive therapy was introduced: methotrexate 7.5 mg a week followed by 10 mg of folic acid two days after. 

## Results

**Fig. 3:**
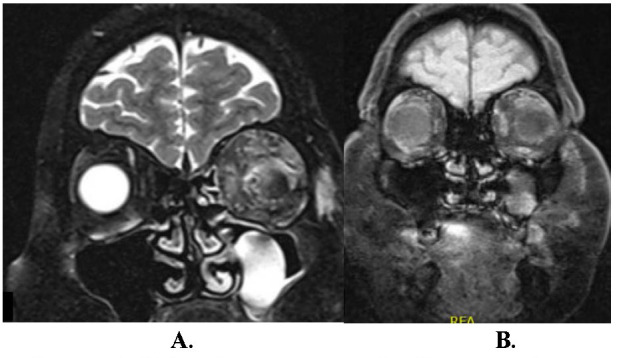
MRI, coronal scans. **A.** Before immunosuppressive therapy - enlargement of left lateral, superior and medial extraocular muscle. **B.** Decrease in thickness after immunosuppressive therapy

Since it takes 4-8 weeks for immunosuppressive therapy to start its effect, she was advised to stay on corticosteroid therapy (1 mg/ kg), tapering the dose gradually. Her local clinical findings, as well as her subjective feeling improved and she was sent home with the following clinical findings: BCVA 0.9, normotension (17 mmHg), decreased left eye proptosis (2 mm), slight left abduction restriction, medial rectus muscle width 1.71 mm shown on ultrasound and MRI (**Fig. 3**).

She was advised to continue local steroid ointment with artificial tears, 60 mg oral prednisolone followed by a weekly tapper and a weekly dose of 7.5 mg methotrexate followed by 10 mg of folic acid 48 hours after. Control laboratory examinations were done three weeks after immunosuppressive therapy was introduced (ESR, CRP, CBC, WBC, urine, liver, and kidney function). Methotrexate dose was increased to 15 mg. Oral steroids were completely discontinued 4 months after she started taking methotrexate. She is currently on 10 mg methotrexate dose, without oral steroids and she does not have any subjective complaints (**Table 1**). After a year of immunosuppressive therapy and after a rheumatology consult, she is planned to have a methotrexate dose tapered to 5 mg for two months and then to discontinue the therapy. 

**Table 1:** Comparison of symptoms before and one year after immunosuppressive therapy introduction

Symptoms	Without immunosuppressive therapy	With immunosuppressive therapy (five months after)
BCVA	HM	0.8-1.0
Intraocular pressure	41 mmHg	17 mmHg
Orbital pain	Yes	Absent
Double vision	Yes	Absent
Motility	Restricted left abduction	Normal
Proptosis (exophthalmometry)	6 mm	1 mm
Palpebral oedema	Severe	Normal
Conjunctiva	Severe chemosis and hyperemia	Normal
Anterior segment	Normal	Normal
Posterior segment	Hypertensive retinopathy gr I	Hypertensive retinopathy gr I
Medial rectus muscle thickness	3.88 mm	1.71 mm
MRI	Enlargement of orbital muscles	Regression of myositis

## Discussion

Diagnosis of ocular myositis can be challenging. Differential diagnosis includes: thyroid orbitopathy (TED), infection, inflammatory reaction, vasculitis, SLE, inflammatory intestine disease, neoplasm, carotid-cavernous fistula, IgG4-related disease, optic neuritis, sarcoidosis, Tolosa-Hunt syndrome, or orbital lymphoma [**5**].

It is important to make a distinction between orbital myositis and TED, since it is the most common cause of orbitopathy. Tendon is usually spared in the thyroid eye disease and it usually involves inferior rectus muscle, unlike medial rectus muscle in orbital myositis [**4**]. Acute pain is also more characteristic for orbital myositis [**6**]. The difference between lymphoproliferative orbit infiltration is that the lymphoma grows slowly and is usually painless. 

The diagnosis of orbital myositis is made using the exclusion method, after the patient’s anamnestic data have been obtained and the physical examination has been conducted. Laboratory tests, including CBC, liver and kidney function tests, thyroid function tests, various antibodies, ESR, CRP, rheumatoid factor, viral markers, chest X-ray, ECG and echocardiography, ultrasonography of thyroid and abdomen and thorax CT should also be performed. Imaging methods include eye ultrasound, which is more frequently available and can be used for follow ups, but also MRI of the orbits with and without contrasts and fat suppression. MRI findings include the thickening of the affected muscle together with the myotendinous insertion. 

Current treatment option and a first line medication for orbital myositis is corticosteroid therapy. The typical dose is 1 mg/ kg/ d for one week, tapering it over 6 to 12 weeks. If it is a very severe case, pulse intravenous methylprednisolone can be administered for three days and then converted to previously mentioned scheme, followed by a slow taper [**4**,**7**]. In a case of recurrence, the dose should again be increased by 5-10 mg per day for a week until the symptoms are improved [**4**]. 

The patient was referred to the rheumatologist due to two exacerbations and for a consultative opinion about immunosuppressive therapy (methotrexate), which is recommended for the patients who fail to respond to steroids, have serious side effects or show relapse. The laboratory work needed for methotrexate administration is ESR, CRP, CBC, WBC, liver and kidney function tests and urine analysis. If the tests are normal, methotrexate is started at a dose 7.5-25 mg/ per week followed by 10 mg of folic acid 48 hours after. It is recommended to control the same lab work three weeks and two months after the therapy was introduced [**5**]. Since it takes 6-8 weeks for immunosuppressive therapy to take its effect, steroid therapy should be continued in the same dose 1 mg/ kg/ d, and to be tapered gradually [**7**]. The main mechanism is inhibition of rapidly dividing cells (leukocytes usually) causing anti-inflammatory effect [**7**]. Since steroids were excluded from her therapy, she lost a lot of weight and her quality of life improved. 

This case helps to create a protocol for corticosteroid sparing agent introduction in orbital myositis with noted recurrences and severe corticosteroid induced side effects. This protocol is used worldwide, but no similar studies were found in Bosnia and Herzegovina, which increases the importance of this case presentation.

## Conclusion

Extraocular myositis is a rare inflammatory orbit disease that needs a prompt diagnosis in order to start the therapy. The diagnosis is made using clinical findings, history of the disease, laboratory exams, ultrasound, and MRI scans, which are all helpful to exclude the diagnosis with similar clinical representation. The first line treatment options are systemic and local corticosteroid therapy. In a case of relapse of the disease after corticosteroid taper or if severe corticosteroid related side effects appear, it is advisable to consider immunosuppressive therapy, if not contraindicated. A suggested algorithm can also be used in other ocular diseases in which systemic corticosteroid treatment is not sufficient or causes severe side effects. A recommendation is to approach each patient individually and to perform a regular monitoring indicated in the algorithm. 

**Conflicts of Interest statement**

The authors declare that there are no conflicts of interest. 

**Informed Consent and Human and Animal Rights statement**

Informed consent has been obtained from the patient included in this study. 

**Authorization for the use of human subjects**

Ethical approval: The case report related to human use complies with all the relevant national regulations, institutional policies, is in accordance with the tenets of the Helsinki Declaration. 

**Acknowledgments**

None. 

**Sources of Funding**

None.

**Financial Disclosure(s)**

None.
